# Severity Analysis of Hazardous Material Road Transportation Crashes with a Bayesian Network Using Highway Safety Information System Data

**DOI:** 10.3390/ijerph19074002

**Published:** 2022-03-28

**Authors:** Ming Sun, Ronggui Zhou, Chengwu Jiao, Xiaoduan Sun

**Affiliations:** 1 Road Safety Research Center, Research Institute of Highway Ministry of Transport, Beijing 100088, China; rg.zhou@rioh.cn (R.Z.); cw.jiao@rioh.cn (C.J.); 2 Department of Civil Engineering, University of Louisiana at Lafayette, Lafayette, LA 70504, USA; xsun@louisiana.edu

**Keywords:** hazardous material road transportation, crash severity, random forest, Bayesian network

## Abstract

Although crashes involving hazardous materials (HAZMAT) are rare events compared with other types of traffic crashes, they often cause tremendous loss of life and property, as well as severe hazards to the environment and public safety. Using five-year (2013–2017) crash data (N = 1610) from the Highway Safety Information System database, a two-step machine learning-based approach was proposed to investigate and quantify the statistical relationship between three HAZMAT crash severity outcomes (fatal and severe injury, injury, and no injury) and contributing factors, including the driver, road, vehicle, crash, and environmental characteristics. Random forest ranked the importance of risk factors, and then Bayesian networks were developed to provide probabilistic inference. The results show that fatal and severe HAZMAT crashes are closely associated with younger drivers (age less than 25), driver fatigue, violation, distraction, special roadway locations (such as intersections, ramps, and bridges), higher speed limits (over 66 mph), midnight and early morning (12:00–5:59 a.m.), head-on crashes, more than four vehicles, fire/explosion/spill, poor lighting conditions, and adverse weather conditions. The overall prediction accuracy of 85.8% suggests the effectiveness of the proposed method. This study extends machine learning applications in a HAZMAT crash analysis, which would help develop targeted countermeasures and strategies to improve HAZMAT road transportation safety.

## 1. Introduction

Because of the fast and widespread expansion of urbanization and industrialization, the demand for hazardous materials (HAZMAT) has increased substantially in recent years. According to the 2017 commodity flow survey [1], over 2.9 billion tons of HAZMAT were moved in the U.S. by all transportation modes in 2017. As one of the most prevalent transportation modes for HAZMAT movement, highway transportation accounted for over 60% of all HAZMAT shipments in the U.S. Although crashes involving HAZMAT are rare events and the number of HAZMAT crashes is relatively small when compared with general traffic crashes, these types of crashes often result in more severe injuries. In 2019, a total of 5005 large trucks were involved in fatal crashes in the United States, of which, 120 (2.4%) were carrying HAZMAT [2]. A ten-year (2012–2021) summary of HAZMAT-related incidents compiled by the U.S. Department of Transportation shows that HAZMAT highway transportation accounted for 100% of the fatalities and 74.02% of injuries [3].

Due to the special physical and chemical properties of HAZMAT, especially the potential risk of explosion, fire, and spillage during an accident, catastrophic crashes involving HAZMAT road transportation are often referred to as low probability with high consequence [4]. These crashes usually lead to tremendous loss of life and property, and the hazards to the environment and public safety should not be neglected. For example, the overall economic effects of class 3 (flammable and combustible liquids) HAZMAT incidents in 1996 were about USD 459 million, including injuries and fatalities, cleanup costs, property damage, evacuation, product loss, traffic delays, and environmental damage [5]. Since HAZMAT road transportation crashes pose a significant threat to the environment, public health and safety, and community well-being, it is important to identify why, where, and how HAZMAT crashes occur.

Researchers often rely on crash data and statistical regression models to identify possible risk factors associated with crash frequency and severity. With the rapid development and widespread application of data mining and machine learning techniques in transportation safety research, more researchers are focusing on data-driven safety analyses to discover the underlying patterns and heterogeneous relationships in the safety data. Concentrated study areas include real-time crash prediction, crash analysis, crash severity analysis, and others. Machine learning approaches (such as neural networks, decision trees, support vector machines, etc.) have been proven to have comparable or superior performance compared with traditional statistical models in crash data analyses [6]. However, a limited number of studies have applied these methods in HAZMAT crash severity prediction. The present study aims to contribute to safer HAZMAT road transportation by focusing on the following objectives:

1. Analyze the crash characteristics and identify the contributing factors that influence HAZMAT road transportation crashes.
2. Quantify the associations between risk factors and different HAZMAT crash severity outcomes.
3. Investigate the role of machine learning approaches in the application of HAZMAT crash severity prediction.

Using five-year (2013–2017) HAZMAT crash data, a two-step machine learning-based method was proposed by combing random forest and a Bayesian network to identify the risk factors and show how these factors affect HAZMAT crash severity. In this study, three injury severity levels (fatal and severe injury, injury, and no injury) were entered as response variables, while driver, road, vehicle, crash, and environmental characteristics were analyzed as the explanatory variables. The proposed approach integrates the variable importance ranking of random forest and the crash severity prediction and interpretability of Bayesian network. Various performance measurements (including accuracy, precision, sensitivity, specificity, F-score, and receiver operating characteristic curves) were utilized for model validation and prediction evaluation. This study will assist transportation management agencies and policymakers in developing targeted countermeasures and strategies to reduce HAZMAT crashes and improve HAZMAT road transportation safety.

## 2. Literature Review

Many studies in the past have conducted HAZMAT crash analyses. Statistical analyses and regression models are the most commonly used approaches to identify the crash characteristics. These methods include crash survey and descriptive analyses [7,8,9], Poisson regression models [10], fixed and random parameters ordered probit models [11,12], ordered logistic models [13,14], and mixed logit models [15]. For example, Oggero et al. [7] investigated 1932 HAZMAT transportation crashes by road and rail from the beginning of the 20th century to 2004. The results showed that the most frequent crash types were releases, followed by fires, explosions, and gas clouds. Shen et al. [9] collected detailed descriptions of 708 HAZMAT road transportation crashes in China from 2004 to 2011. The findings indicated that freeways, early morning (4 a.m. to 6 a.m.) and midday hours (10 a.m. to 12 p.m.), human-related errors, and vehicle-related defects were the contributing factors of these crashes. Some studies also utilized discrete choice models to further quantify the relationship between risk factors and HAZMAT crash severity. Uddin and Huynh [10] analyzed 1173 HAZMAT crashes in California from 2005 to 2011. Fixed and random parameters ordered probit models were developed to investigate the risk factors that affect crash injury severity. The results showed that male occupants, truck drivers, crashes in rural areas, dark–unlighted conditions, dark–lighted conditions, and weekdays were associated with a higher likelihood of major injuries. Xing et al. [12] conducted a comprehensive analysis of 1721 HAZMAT crashes in China between 2014 and 2017. By considering the unobserved heterogeneity in the crash data, a random-parameters ordered probit model was developed to investigate the impacts of contributing factors on the HAZMAT crash severity. The following variables were found to be associated with increased injury severity: type of HAZMAT (such as compressed gas, explosive, and poison), driver behavior (including misoperations, fatigue, and speeding), roadway features (tunnel, slope, county road, and dry road surface condition), winter, dark lighting conditions, more than two vehicles, rear-end crashes, and explosions.

Although conventional statistical methods have been proven effective in examining the relationships between HAZMAT crash severity and explanatory variables, they cannot reveal the underlying patterns and interplay of various factors [16]. In recent years, machine learning techniques, such as Bayesian networks [16,17,18,19,20], clustering [21,22], support vector machines [23], decision trees [4,24], random forests [25,26], and association mining rules [27,28] have been widely used for crash data analysis. These non-parameter approaches do not require assumption among explanatory variables, and they have been identified as having greater flexibility in supporting in-depth crash analysis and safety decision-making. For example, Ma et al. [16] collected 839 HAZMAT crashes in China from 2015 to 2016 to explore the impact of risk factors on crash occurrence. The study found that the Bayesian network’s posterior probability can serve as an effective tool for identifying the important factors (including HAZMAT type, crash location, driver, environment, and vehicle factors) and the combination of crash factors. Zhao et al. [18] applied Bayesian networks to prioritize the factors contributing to HAZMAT crashes. Human factors, the transport vehicle, and facilities, and packing and loading of the HAZMAT were the three most influential factors in HAZMAT transportation crashes. By comparing four statistical and machine learning methods for crash severity prediction, Iranitalab and Khattak [22] concluded that the nearest neighbor classification has the best prediction performance in total and more severe crashes. Hong et al. [27] applied the association mining rules to discover the risk factors contributing to crashes involving HAZMAT vehicles on expressways. The results illustrated that male drivers, single-vehicle crashes, daytime, clear weather conditions, and mainline segments were closely related to HAZMAT vehicle-involved crashes.

However, despite the machine learning methods mentioned above yielding relatively good performance results on crash severity modeling, HAZMAT injury severity prediction was not a key focus. Some of the approaches may not explain the relationship between contributing factors and reflect the underlying crash mechanism. The application of Bayesian networks in causal analysis can provide probability inference for crash severity under multiple factors. This study proposed a two-step machine learning-based approach for HAZMAT crash severity analysis and prediction. Random forest was used as a preliminary tool to rank the importance of influencing factors on HAZMAT crash severity. Bayesian networks were used to further identify the relationship between risk factors and HAZMAT crash severity, predict the probability distribution of severity outcomes, and provide a decision-making basis for HAZMAT crash risk reduction.

## 3. Materials and Methods

### 3.1. Crash Data

Five-year crash data (2013–2017) involving HAZMAT road transportation in North Carolina, Ohio, and Washington were retrieved from the Highway Safety Information System (HSIS). HSIS is a roadway-based system that contains highway patrol-reported data on a wide range of crashes and other relevant information, including occupants, vehicles, roadways, and traffic volume (annual average daily traffic, AADT) involved in the crash. Data from the various datasets (crash data, roadway data, and vehicle data) were matched and merged with the unique crash identification number, county route number, and milepost information.

The crash severity was recorded using a KABCO injury scale in the HSIS database:

- Fatality (K);
- Severe or incapacitating injury (A);
- Evident or non-incapacitating injury (B);
- Possible or compliant injury (C);
- Property damage only (O).

In this study, severity levels K and A were combined as fatal and severe crashes, severity levels B and C were grouped as injury crashes. These data combinations ensured that each injury severity level had a sufficient number of observations. Researchers frequently use similar strategies to ensure sufficient sample sizes for data analyses and model estimations [11,15,21,29,30].

Based on the availability and suitability to explain the injury severity of HAZMAT road transportation crashes, a total of 25 potential influencing factors were selected as explanatory variables. These factors were further classified into five categories: driver, road, vehicle, crash, and environmental characteristics. The crash records with missing values for the investigated factors were removed from the final dataset for model development.

A total number of 1610 HAZMAT crashes were included in the final dataset. Based on the previously defined three injury severity levels in this study, there were 106 (6.6%) fatal and severe injury crashes, 405 (25.2%) injury crashes, and 1099 (68.2%) no injury crashes. Table 1 provides an overview of the descriptive statistics of the HAZMAT road transportation crashes.

### 3.2. Random Forests

Random forest is a machine learning method that ensembles a large number of decision trees. It is used to solve both classification and regression problems. A bootstrap sample and an out-of-bag (OOB) validation strategy are used to train each tree. The optimal variables at each split are identified from a random subset of all variables. The global prediction of random forests is based on a majority vote on the predictions of each classification tree. It is a popular method for evaluating the significance of explanatory variables and variable selection in recent years [20,31,32]. Compared to many other commonly used classifiers, this method has proved to be performed very well and robust to overfitting [31]. Random forests were applied in this study to prioritize the importance of influencing factors on HAZMAT crash severity and reduce the redundancy of variables.

Suppose there are M input variables in the original dataset D. $X=\left\{x_{1}{,x}_{2},\;\cdots {,\;x}_{n}\right\}$ is a training set and $Y=\left\{y_{1},y_{2},\;\cdots {,\;y}_{n}\right\}$ are the responses. Bootstrapping refers to the process of selecting a random sample of m variables repeatedly (B times) with the training set (m < M) being replaced. The remaining out-of-bag (OOB) samples of the training set were not used in the tree generation but to obtain an estimation of unbiased errors. Train a classification tree $f_{b}$ based on the selected random sample $X_{b},\;Y_{b}$ and choose the best split variable. The prediction error $\hat{f}$ can be calculated by averaging the predictions from all the individual trees:

(1) $$\hat{f}=\frac{1}{B}\sum_{b=1}^{B}f_{b}(x^{\prime })$$

Each tree classifier iterates over every unutilized feature in the set D during each training process and generates a classification result for the target variables based on the OOB samples through majority voting. If a single or a few variables are particularly strong predictors for the response variables, they will be selected in many tree classifiers. The random forests calculate the feature importance by rearranging the errors before and after classification. The average of a variable’s overall decrease in node impurity is defined as the mean decrease in Gini. In the random forests, it is weighted by the proportion of samples at the node for each individual decision tree. A higher value of mean decrease in Gini indicates higher variable importance. The Gini impurity and mean decrease in Gini can be calculated as follows:

(2) $$\mathrm{Gini}=1-\sum_{i=1}^{C}p_{i}^{2}$$

(3) $$\mathrm{VI}(x_{j})=\frac{1}{B}(1-\sum_{b=1}^{B}\mathrm{Gini}{\left(j\right)}^{b})$$

The number of classes in the target variable is represented by C, and the proportion of this class is represented by $p_{i}$, Gini(j) represents the Gini index for variable $x_{j}$.

### 3.3. Bayesian Networks

Bayesian networks integrate principles from graph theory and Bayes’ probability theory to extract correlations between independent and dependent parameters for probabilistic inference. As a result, Bayesian networks can model intercorrelated independent parameters and explain the heterogeneous impacts on various HAZMAT crash injury severity levels through variable changes. A directed acyclic graph (DAG) represents a joint probability distribution over a set of parameters in a Bayesian network structure [33]. It captures the statistical relationships and extracts all interactions between independent and dependent parameters. A set of variables $V=\left\{x_{1}{,x}_{2},\;\cdots {,\;x}_{n}\right\},\;n>1$ is represented by nodes in a DAG, with the edges representing the direct dependencies between these variables. $B_{p}=\left\{p\left(x_{i}|pa\left(x_{i}\right){,x}_{i}\in V\right)\right\}$ is a set of probability tables. In Bayesian networks, $pa\left(x_{i}\right)$ represents the set of antecedents or parents of $x_{i},$ (i=1,2, ⋯, n). The joint probability distribution over V in Bayesian networks can be calculated as:

(4) $$P\left(x_{1}{,x}_{2},\cdots {,x}_{n}\right)=\prod_{i=1}^{n}p\left(x_{i}|\mathrm{pa}\left(x_{i}\right)\right)$$

Each node in the graph represents a conditional probability table with the state of the variable. Given the states of a node’s parents, this table contains the node probabilities of being in a specific state. Bayesian networks are usually unknown and need to be developed via expert knowledge or a given dataset.

#### 3.3.1. Structure Learning of Bayesian Networks

The K2 algorithm is utilized to develop the original DAG of Bayesian networks for parameter learning. Based on a predetermined order of nodes, the K2 algorithm heuristically searches and recovers the most likely underlying structure. This algorithm starts with the assumption that a node does not have any parents. The addition of the parents keeps on when it increases the likelihood of the resulting structure. This process continues until adding of a single parent no longer increases the probability. The following equation describes the K2 algorithm [34]:

(5) $$P\left(G,D\right)=P(G)\prod_{i=1}^{n}\prod_{j=1}^{q_{i}}\frac{\left(r_{i}-1\right)!}{\left(N_{\mathrm{ij}}{+r}_{i}-1\right)!}\prod_{k=1}^{r_{i}}N_{\mathrm{ijk}}!$$

D is a random data sample $D=\left\{d_{1}{,d}_{2},\cdots {,d}_{n}\right\}$, G is the Bayesian network structure, P(G) is the prior probability for a structure G, n is the numbers of nodes, $q_{i}$ is the states of ith node’s parents, $r_{i}$ is the ith node’s mutual exclusive states, and $N_{\mathrm{ijk}}$ denotes the ith node is in the kth state while its parents are in the jth state, where $N_{\mathrm{ij}}=\overset{r_{i}}{\underset{k=1}{\sum }}N_{\mathrm{ijk}}$.

#### 3.3.2. Parameter Learning of Bayesian Networks 

The expectation–maximization (EM) algorithm is a widely applicable method for iteratively calculating maximum likelihood (ML) estimates. It can be used to solve various problems with incomplete data or a small sample size. In particular, the EM algorithm greatly simplifies the problem of fitting finite mixture models through ML. The EM algorithm has many attractive features, such as numerical stability, simplicity of implementation, and reliable global convergence [35]. Past studies [36,37] have shown its effectiveness in Bayesian network parameter learning. In general, the EM algorithm consists of three steps:

1. Assign an initial value for the model parameter.
  Given the observation variable $Y=\left\{Y_{1}{,Y}_{2},\cdots {,Y}_{n}\right\}$, the hidden variable $Z=\left\{Z_{1}{,Z}_{2},\cdots {,Z}_{n}\right\}$, P(Y,Z|θ) is the joint distribution, P(Z|Y,θ) is the conditional probability distribution. θ represents the maximum likelihood parameter, which is quantified by the log-likelihood function:
  (6) $$L_{D}\left(\theta \right)=\log\sum_{Z}P(Y,Z|\theta )$$
2. E-step: in the ith iteration, the value of the current model parameter becomes $\theta_{i}$, given the observed data Y and $\theta_{i}$, calculate the conditional expectation of the log-likelihood function.
  (7) $$Q\left({\theta |\theta }_{i}\right){=E}_{\theta_{i}}[\mathrm{logP}(D|\theta )|\theta_{i},Y]$$
3. M-step: based on the joint probability distribution estimation in the E-step, find an updating parameter $\theta_{i+1}$ that maximizes the expected log-likelihood.
  (8) $$\theta_{i+1}=\underset{\theta }{\mathrm{argmax}}Q(\theta |\theta_{i})$$

Repeat steps 2 to 3 until the model coverages. Each iteration ensures that the likelihood will increase, and the algorithm eventually converges to a local maximum of the likelihood function.

## 4. Results and Discussions

### 4.1. Variable Selection

**Figure 1:**
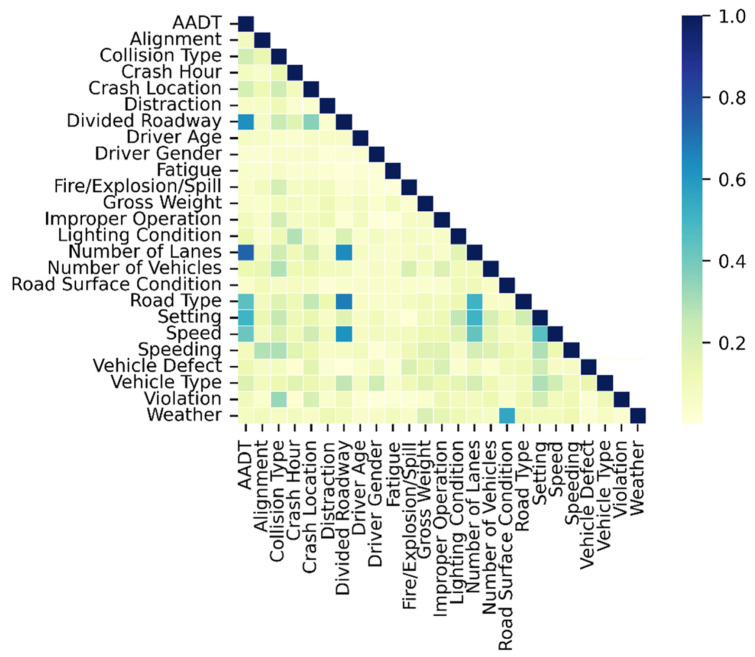
Variable correlation matrix.

Correlation analysis was conducted with Cramer’s V statistics for variables listed in Table 1. Cramer’s V statistics measure the strength of association among categorical variables, ranging from 0 to 1 [38]. A higher value indicates a stronger association. Calculation of Cramer’s V statistics was completed in SPSS Statistics 27. The variable correlation matrix is shown in Figure 1. Divided roadway and number of lanes were highly associated with AADT (the correlation coefficients are 0.628 and 0.737, respectively, *p*-values are less than 0.05). Number of lanes, road type, and speed were strongly associated with divided roads with Cramer’s V statistics of 0.644, 0.676, and 0.638, respectively (*p*-values are less than 0.05). There was a significant association between road surface conditions and weather (0.601, *p*-value is less than 0.05). Previous studies have also confirmed this relationship [39,40].

**Figure 2:**
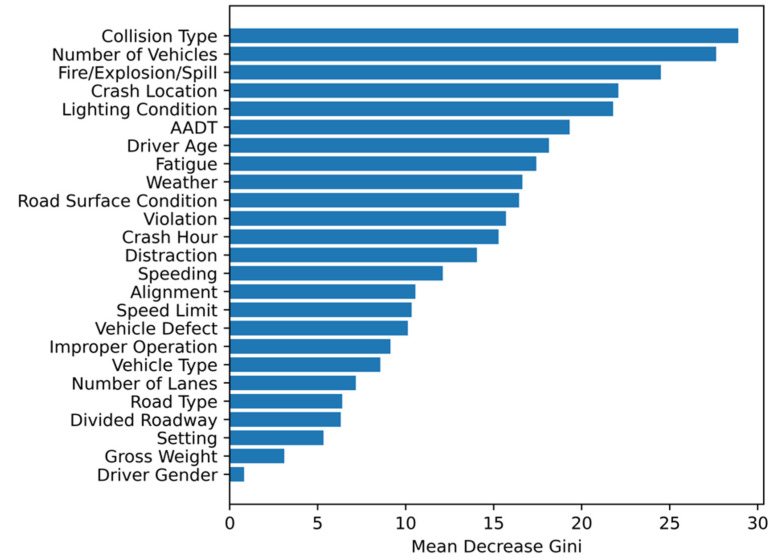
Variable importance ranking using random forests.

The variable importance ranking obtained by random forests is shown in Figure 2. The collision type (28.90), number of vehicles (27.64), the occurrence of fire/explosion/spill (24.49), crash location (22.08), and lighting condition (21.78) were identified as the five most crucial variables. Driver gender and gross vehicle weight have a limited impact on the HAZMAT crash severity as these two factors have the mean decrease Gini values of 0.80 and 3.10, respectively. The top 18 factors accounted for 90% of cumulative mean decrease Gini values. Based on the correlation analysis results and the variable importance ranking, number of lanes, road type, divided roadway, road surface conditions, setting, vehicle gross weight, and driver gender were eliminated from the potential risk factors due to their limited impacts on the HAZMAT crash severity, and to avoid collinearity between the independent variables. Therefore, 18 factors (collision type, number of vehicles, fire/explosion/spill, crash location, lighting condition, AADT, driver age, fatigue, weather, vehicle defect, violation, hour, distraction, speeding, alignment, speed limit, improper operation, vehicle type) were selected as influencing factors for Bayesian network structure learning, model development, and conditional probability inference.

### 4.2. Bayesian Network Development

**Figure 3:**
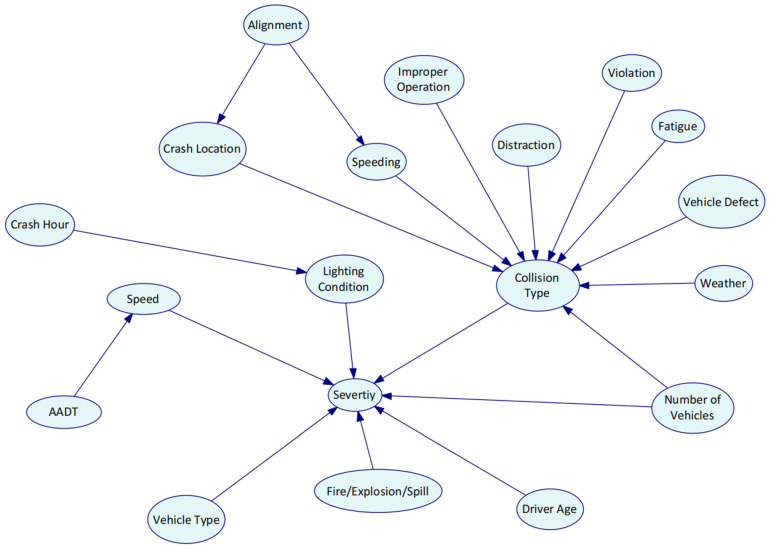
The Bayesian network structure development.

**Figure 4:**
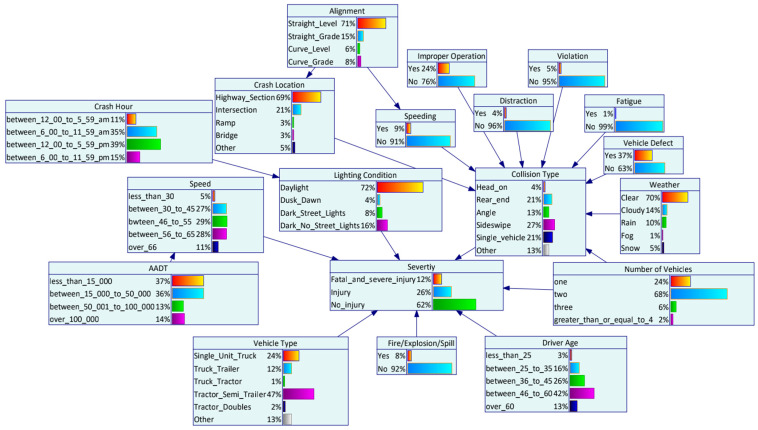
The Bayesian network parameter learning.

Genie 2.0 software was utilized to establish the Bayesian network structure and estimate the parameters with the EM algorithm. The initial DAG was developed based on the 18 significant variables identified by random forests. To investigate the interdependency between contributing factors and HAZMAT crash injury severity, the initial DAG was completed using the K2 algorithm. Modification of the Bayesian network structure was performed based on expert knowledge afterward. Eight experts within the HAZMAT road transportation field (three experts from universities, two experts from engineering consulting companies, and three experts from road safety research centers) filled in a questionnaire. The initial Bayesian network structure developed by the K2 algorithm based on the collected data were distributed to the experts. The relationship between HAZMAT crash severity and the risk factors was reviewed by the experts. A direction is considered effective if more than five experts confirm the causality. By collecting the opinions from experts and updating the DAG accordingly, the final structure was determined. Figure 3 shows the graphical structure of Bayesian networks. The nodes reflect variables, and the edges reflect the direct dependencies between the target node (HAZMAT crash injury severity) and the variables in the graph. The prior probability distribution $P\left(x_{i}\right)$ of each variable after parameter learning is shown in histograms of Figure 4.

### 4.3. Bayesian Network Inference Results and HAZMAT Crash Characteristics Analysis

Based on the developed Bayesian network model and the inference results, the probability distributions of HAZMAT crash occurrence are illustrated in Table 1. Setting evidence of the variables used in the development of the Bayesian network model provides indications to the values of variables contributing to HAZMAT crash occurrence and injury severity. For each variable in the Bayesian network model, the probability of a value for a specific variable was set to 1.0 (also referred to as setting evidence), while the other values of the same variable were assigned to 0.0. As a result, the conditional probability tables of Bayesian networks can be used to calculate the related probability of HAZMAT crash injury severity, which could identify the most significant contributing values or variables to the HAZMAT road transportation crash injury severity outcomes and the underlying crash mechanism. Given HAZMAT crash occurrences, Table 1 shows the Bayesian network probability inference results for the contributing factors’ influence on the three levels of crash injury severity (fatal and severe injury, injury, and no injury).

#### 4.3.1. Driver Characteristics

The results shown in Table 2 indicate that assigning the value “less than 25” of the variable “driver age” a probability of 1.0, the probability of fatal and severe injury HAZMAT crashes changes from 0.1239 to 0.2187 (an increase of 76.5%), while the probability of injury crashes changes from 0.2602 to 0.3348 (an increase of 28.7%). The younger drivers (age less than 25) were found to be suffered more fatal and severe injuries in HAZMAT crashes. Older drivers (over 60) were also found to be more likely to experience fatal and severe injuries of HAZMAT crashes with a probability of 0.1527 (23.3% increase). These findings are consistent with findings from previous research. According to Tavris et al. [41] and Ma et al. [16], younger drivers were substantially more prone to involve in severe and fatal HAZMAT crashes. Drivers between 55 and 65 years old were found to be more likely experience severe injuries [42].

**Figure 5:**
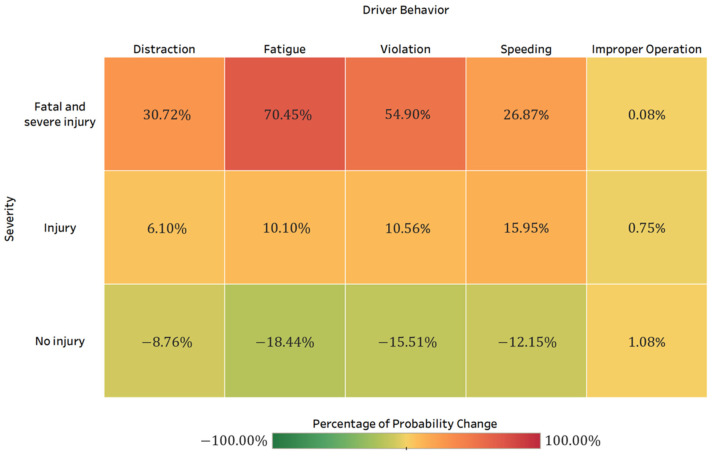
Percentage of probability change for HAZMAT crash severity when setting evidence for driver behavior factors.

Driver behavior was found to significantly impact the HAZMAT crash severity. As shown in Table 2 and Figure 5, the fatal and severe injury crash probability attributed to driver fatigue is the highest among other driver behavior-related factors, changing from 0.1239 to 0.2112 (an increase of 70.45%). The model results also illustrate that violation (the posterior probability is 0.1920, increasing by 54.9%), distraction (the posterior probability is 0.1620, increasing by 30.7%), and speeding (the posterior probability is 0.1572, increasing by 26.9%) are closely associated with fatal and severe injuries in HAZMAT crashes. In comparison, the fatal and severe injury crash probability caused by improper operation is relatively lower. Previous researchers have found the association between driver behavior and crash occurrence [13,16,18,27]. Xing et al. [12] and Luo et al. [19] both found that driver fatigue and speeding significantly impact the HAZMAT crash injury severity. One of the most prevalent causes of traffic crashes related to behavioral error is driver inattention [43]. It is associated with impaired driving performance and significant deficiencies in cognitive performance, both of which could have a negative impact on road safety. Compared with passenger vehicle drivers, professional drivers are more frequently exposed to longer driving distances and travel time, leading to a higher possibility of safety risk for distraction and fatigue [44,45].

#### 4.3.2. Road Characteristics

The results shown in Table 2 indicate that by assigning the value “curve-grade” of the variable “alignment” a likelihood of 1.0, the probability of fatal and severe injury HAZMAT crashes changes from 0.1239 to 0.1318 (an increase of 6.4%). For HAZMAT crashes occurring on locations other than highway sections, the Bayesian network model results indicate that intersections, ramps, bridges, and other special road features increased the likelihood of fatal and severe injuries by 21.3%, 32.2%, 27.6%, and 20.2%, respectively. Higher exposed crash risks can be explained by the presence of additional interference factors, conflict points, and potential risks (such as pedestrians, merging and diverging maneuvers, vision obstruction).

As expected, higher speed limits (over 66 mph) were found to be closely related to increased fatal and severe injury probabilities of HAZMAT crashes occurrence. The posterior probability is 0.1533 (increasing by 23.7%). An interesting finding is that low speed limits (less than 30 mph) also increase the likelihood of fatal and severe HAZMAT crashes with a posterior probability of 0.1399 (an increase of 12.9%). One possible explanation could be that the relatively lower speed limits are associated with urban streets, which involve a more complex road environment and more interference factors.

#### 4.3.3. Crash Characteristics

**Figure 6:**
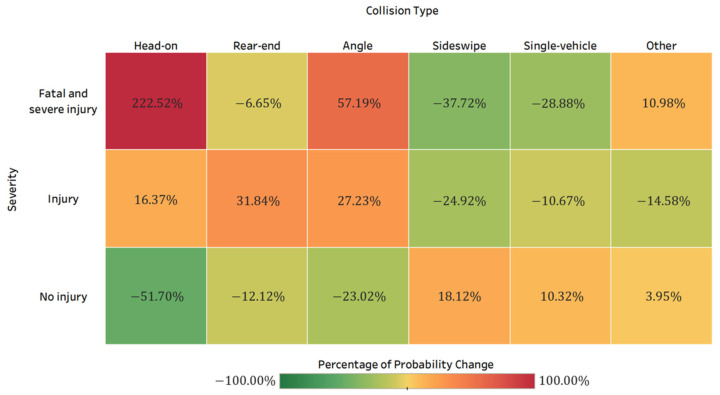
HAZMAT road transportation collision type proportions by crash severity.

Table 1 and Figure 6 display the HAZMAT road transportation collision type proportions across different crash severity levels. Among all HAZMAT fatal and severe injury crashes and injury crashes, head-on crashes accounted for the highest percentage (41.9% and 38.7%, respectively). As confirmed by Bayesian network models, head-on crashes increase the likelihood of fatal and severe injury HAZMAT crashes by 222.5% (the posterior probability is 0.3997), while it is 16.4% (the posterior probability is 0.3028) for injury crashes. With a posterior probability of 0.1948, angle crashes were also found to be highly related to fatal and severe injury HAZMAT crashes (increasing by 57.2%). On the contrary, rear-end and sideswipe crashes decrease the probability of fatal and severe injuries by 6.7% and 37.7%, respectively. When there was only one vehicle involved in a HAZMAT crash, the probability of fatal and severe injury HAZMAT crashes decreased by 28.9%. Head-on crashes were found to be more harmful than angle crashes in past studies [17], as these types of crashes frequently resulted in fatalities or severe injuries.

**Figure 7:**
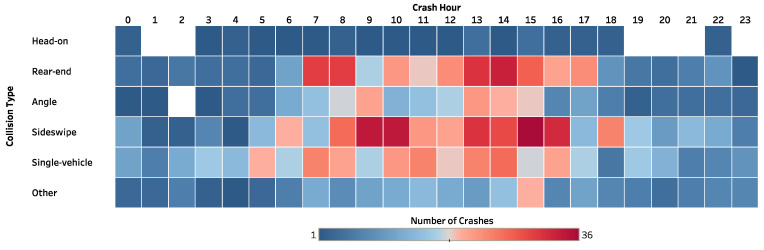
The number of HAZMAT road transportation crashes by collision type and crash hour.

Figure 7 illustrates the crash hour distribution by collision type. The color intensity represents the number of crashes. A substantial number of crashes occurred during daytime (7:00 a.m.–4:00 p.m.), particularly for rear-end and sideswipe crashes. However, based on Bayesian network model results, when setting a probability of 1.0 to the value “12:00–5:59 a.m.” of the variable “crash hour,” the probability of fatal and severe injury HAZMAT crashes changes from 0.1239 to 0.1736 (an increase of 40.10%), following by nighttime (6:00–11:59 p.m.) with a posterior probability of 0.1605 (increasing by 29.5%). Although many crashes occur during the daytime, crashes during midnight are more likely to cause fatal and severe injuries. According to past studies [4,9,12,46], poor visibility at night, fatigue, and distraction could be some of the potential causes.

Regarding the crash consequences, the posterior probability of fire/explosion/spill reached 0.2396, increasing the probability of fatal and severe HAZMAT crashes by 93.3%. This result can be explained by the fact that the release of HAZMAT could cause immediate poisoning and suffocation due to their properties, especially in urban areas with high population density. This finding was also confirmed by other studies on HAZMAT crashes [12,16].

#### 4.3.4. Vehicle Characteristics

When it comes to the total number of vehicles involved in the HAZMAT crashes, more than or equal to four vehicles increase the probability of fatal and severe injury crashes by 141.7% (the posterior probability is 0.2995). It is reasonable that more vehicles would result in more persons being involved in crashes, causing more injuries. The results are consistent with the findings of [12,16,47]. Truck/trailer increases the probability of fatal and severe crashes by 6.3%. Vehicle defects were not found to be highly associated with the likelihood of fatal and severe injury crashes in this study.

#### 4.3.5. Environmental Characteristics

The Bayesian network model results indicate that the likelihood of fatal and severe injury HAZMAT crashes increases under dusk/dawn (80.7%), dark-lighted (33.1%), and dark-unlighted (42.8%) conditions. These findings regarding lighting conditions are in line with previous crash studies [17]. In addition, adverse weather conditions were associated with an increased probability of fatal and severe injury HAZMAT crashes. Specifically, snow increases the likelihood of fatal and severe injuries by 10.1%, with a posterior probability of 0.1364. Poor visibility and the relatively lower road friction coefficient could cause crashes and severe injuries.

### 4.4. Model Validation

To measure the performance of the developed Bayesian networks, a confusion matrix for multiclass classification was used to calculate the performance evaluation indicators, including accuracy, precision, sensitivity, specificity, F-score, and ROC area. Table 3 displays the confusion matrix, and the model performance measurements are defined as follows:

(9) $$\mathrm{Accuracy}=\frac{\mathrm{TP}+\mathrm{TN}}{\mathrm{TP}+\mathrm{FP}+\mathrm{FN}+\mathrm{TN}}$$

(10) $$\mathrm{Precision}=\frac{\mathrm{TP}}{\mathrm{TP}+\mathrm{FP}}$$

(11) $$\mathrm{Sensitivity}=\frac{\mathrm{TP}}{\mathrm{TP}+\mathrm{FN}}$$

(12) $$\mathrm{Specificity}=\frac{\mathrm{TN}}{\mathrm{FP}+\mathrm{TN}}$$

(13) $$F-\mathrm{score}=\frac{2\times \mathrm{precison}\times \mathrm{sensitivity}}{\mathrm{precison}+\mathrm{sensitivity}}$$

where true positive (TP) is an indicator that quantifies the number of true positive cases that are accurately detected. For instance, it is the number of fatal and severe injury HAZMAT crashes that are correctly identified as fatal and severe injury crashes in our study. True negative (TN) is an indicator that quantifies the number of true negative cases that are accurately detected, for example, the number of non-fatal and severe crashes that are correctly identified as no injury or injury. False positive (FP) refers to the number of predicted instances that are wrongly labeled as positive cases when they are actually negatives, whereas False negative (FN) refers to the number of predicted instances that are wrongly labeled as negative cases when they are actually positives.

The proportion of correctly classified cases is defined as accuracy, whereas sensitivity is the proportion of cases accurately detected as positive cases out of all true positive cases, and specificity represents the proportion of cases accurately detected as negative cases out of all true negative cases. Nevertheless, there is a tradeoff between sensitivity and specificity, so we calculated the weighted average of sensitivity and specificity, known as F-score. The receiver operating characteristic (ROC) area is another commonly used and effective tool for measuring the overall performance of classification models. The sensitivity (true positive rate) versus 1-specificity (false positive rate) is represented by ROC curves, with a maximum of 1.0 indicating a perfect test and a value of 0.50 indicating a meaningless test.

This study applied a k-fold cross-validation strategy to validate the developed Bayesian network models. The k-fold cross-validation method divides the data into k smaller sets, with (k − 1) of the sets as training data. The remaining sets are utilized as testing data to perform the model validation. This iteration is repeated for each of the k subsets, resulting in each subset being used (k − 1) times as training subset and exactly once as validation subset. Ultimately, we averaged the classification accuracy and error rates over all k experiments to calculate a single performance metric. The value of k is usually 10 in k-fold cross-validation, which is adequate for most classification problems. In this study, five-fold cross-validation was used to reduce computation time.

**Figure 8:**
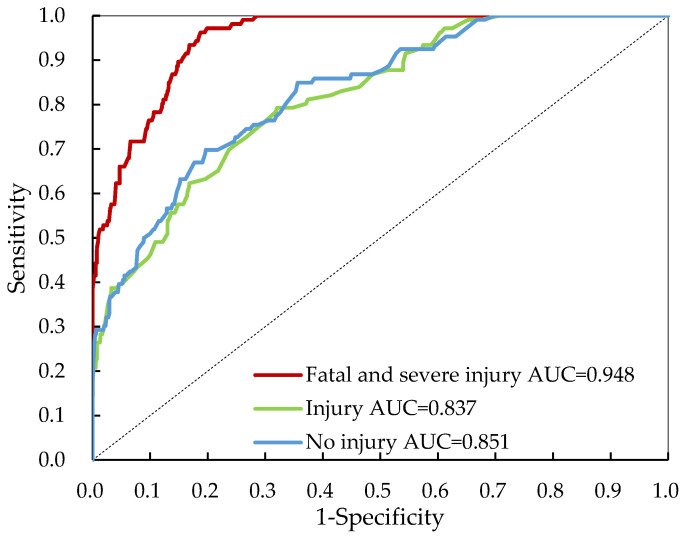
ROC curve for the Bayesian network model evaluation.

The overall Bayesian network estimation results are shown in Table 4. The accuracy values are 96.4% for fatal and severe crashes, 85.9% for injury crashes, and 85.0% for no injury crashes, respectively. In terms of sensitivity, it ranges from 69.4% (injury crashes) to 89.8% (no injury crashes). All the HAZMAT severity categories show an acceptable model prediction performance for predicting positive instances correctly in all actual positive instances. The proportion of instances correctly predicted as negative in all actual negative instances is defined as specificity. The results show that the Bayesian network model can classify 97.1% of fatal and severe injury crashes correctly, but its ability to classify no injury crashes is relatively poor. Since the dataset is distributed in an imbalanced way, the accuracy, sensitivity, or specificity alone is somewhat misleading. This study also used the area under ROC to measure the overall model performance. The highest ROC index is 94.8%, achieved by the Bayesian network for fatal and severe injury crashes classification as shown in Figure 8. The ROC index is 83.7% and 85.1% for classifying injury crashes and no injury crashes, respectively. All three levels of crash severity were successfully classified. The obtained ROC results demonstrate that the proposed combination of random forest and Bayesian network approaches accurately classifies HAZMAT crash severity.

### 4.5. Implications of Study Findings

Based on the analysis results, this study highlights the need for targeted countermeasures for various risk factors, including the driver, road, vehicle, crash, and environmental characteristics. Transportation management agencies and policymakers may directly use the results presented in Table 2 to reduce HAZMAT crashes and improve HAZMAT road transportation safety. The following recommendations are provided for a decision-making basis:

- Enhance safety education, training, and driver monitoring to reduce inappropriate driving behavior, especially fatigue driving, distraction driving, violation, and speeding.
- Improve traffic management and supervision of special locations (such as intersections, ramps, and bridges) with high crash probability on HAZMAT road transportation route. Dynamic monitoring and rapid response system should be established to reduce potential crash risks in these locations.
- HAZMAT carriers may prioritize the fleet management, scheduling, and routing options to avoid the time of midnight or early morning, poor lighting conditions, and adverse weather for potential increased crash risks.
- Spatially or temporally separate HAZMAT vehicles and other vehicles may effectively reduce the fatal and severe injury crash probability, especially for head-on crashes. A dedicated lane or designated time period may help reduce multi-vehicle crashes and prevent post-crash HAZMAT release, fire, and explosion risk.
- Traffic management, safety education, and enforcement strategies must collaborate to ensure safe HAZMAT road transportation.

The results obtained in this study also show that in addition to the prediction accuracy, the combination of random forest and Bayesian networks can effectively quantify the statistical relationships between HAZMAT crash severity and contributing factors. In recent years, researchers have paid more attention to the prediction performance of machine learning-based approaches compared with conventional statistical models in crash data analysis. However, interpretability and lack of transparency have been critical issues. This study contributes to the state of literature by revealing how the risk factors associated with different levels of injury severity of HAZMAT crashes and by extending the application of machine learning approaches in HAZMAT road transportation safety analysis.

## 5. Conclusions

HAZMAT road transportation crashes pose significant safety risks on public life, properties, and the environment. Identifying the characteristics of HAZMAT crashes and the risk factors that contribute to crash severity is crucial for HAZMAT crash reduction and safety improvement. Using five-year crash data (N = 1610) from the HSIS database, this paper proposes a two-step machine learning-based approach by combing random forest and Bayesian network to quantify the statistical relationship between three HAZMAT crash injury severity outcomes (fatal and severe injury, injury, and no injury) and contributing factors. These factors include driver, road, vehicle, crash, and environmental characteristics. Random forest ranks the importance of risk factors, and Bayesian networks are developed to reveal the interdependency between the examined variables and provide probabilistic inference. The main findings are as follows:

- Driver behaviors have a significant influence on the HAZMAT crash injury severity. It is alarming to find that fatigue, violation, distraction, and speeding increase the probability of fatal and severe injury by 70.45%, 54.9%, 30.7%, and 26.9%, respectively. It implies the importance of safety education programs and enhanced driver monitoring and warning techniques to reduce risky driving behaviors.
- Special roadway locations (such as intersections, ramps, and bridges) and higher speed limits (over 66 mph) pose increased fatal and severe HAZMAT crash risks. Identifying zones with a higher possibility of crash risk, enhancing transportation management, and supervision in such locations, and setting up a dynamic response system might reduce the occurrence of severe injury HAZMAT crashes.
- Among all HAZMAT collision types, head-on crashes increase the probability of fatal and severe injury crashes by 222.5%. In contrast, sideswipe and single-vehicle crashes significantly reduce the likelihood of fatal and severe injuries. When a HAZMAT crash involves fire/explosion/spill, the probability of fatal and severe HAZMAT crashes increases by 93.3%. In terms of the total number of vehicles involved in the HAZMAT crashes, more than or equal to four vehicles would result in an increase of 141.7% of the probability of fatal and severe injury crashes.
- Although 74.5% of the HAZMAT crashes occurred during daytime, crashes that occur during midnight, early morning (12:00–5:59 a.m.), and night (6:00–11:59 p.m.) are more likely to cause fatal and severe injuries. Poor lighting conditions (dusk/dawn, dark-lighted, and dark-unlighted) and adverse weather conditions are closely associated with fatal and severe HAZMAT crashes.
- By using a five-fold cross-validation strategy, the combined random forest and Bayesian network models can effectively predict HAZMAT crash injury severity with an overall accuracy of 85.8%. Specifically, the relationship between variables is inherently considered in the Bayesian network. The proposed model can provide reliable crash severity prediction performance and reveal the complex interdependency between the contributing factors.

This study provides an insight into the HAZMAT crash characteristics, discovers how the contributing factors affect HAZMAT crash injury severity, and investigates the underlying crash mechanism behind the HAZMAT crash data. The proposed method presents a potential application of machine learning-based approaches in HAZMAT road transportation safety analysis with relatively satisfying crash severity prediction accuracy and interpretability. In addition, the study demonstrates a clear need to develop targeted HAZMAT crash countermeasures. Enhancing driver safety awareness and education, avoiding driver fatigue, distraction, and other improper driving behavior through in-cabin detection devices and driving monitoring and warning systems, improving traffic management on special locations with high crash probability on HAZMAT road transportation routes, and promoting enforcement strategies should be implemented to reduce HAZMAT crashes and ensure safe HAZMAT road transportation environments.

This study has several limitations that should be considered and improved in the future. Firstly, the investigated contributing factors were limited to those available in the HSIS database from 2013 to 2017. There were many missing values or attributes in the crash records in different states. Secondly, the impact of releasing hazardous materials into the surrounding population was not considered in this study. The population along the HAZMAT transportation route should be included in a future study, and additional data are needed to examine the environmental damage related to HAZMAT crashes. Thirdly, the proposed model was developed and validated on the same dataset from HSIS. Future work should also focus on model transferability to other crash databases.

## Figures and Tables

**Table 1:** Characteristics of HAZMAT road transportation crashes by severity.

Variables	Number of Crashes	Fatal and Severe Injury Crashes	Injury Crashes	No Injury Crashes	Percentage of Total
*Driver characteristics*					
**Driver age**					
Less than 25	49	10.20%	30.61%	59.19%	100%
25–35	262	8.02%	21.76%	70.22%	100%
36–45	415	5.78%	27.23%	66.99%	100%
46–60	682	5.87%	25.22%	68.91%	100%
over 60	202	7.92%	23.76%	68.32%	100%
**Driver gender**					
Male	1546	6.79%	24.97%	68.24%	100%
Female	64	1.56%	29.69%	68.75%	100%
**Distraction**					
Yes	62	1.61%	43.55%	54.84%	100%
No	1548	6.78%	24.42%	68.80%	100%
**Fatigue**					
Yes	10	10.00%	40.00%	50.00%	100%
No	1600	6.56%	25.06%	68.38%	100%
**Improper operation**					
Yes	379	5.01%	24.27%	70.72%	100%
No	1231	7.07%	25.43%	67.50%	100%
**Speeding**					
Yes	140	6.43%	35.71%	57.86%	100%
No	1470	6.60%	24.15%	69.25%	100%
**Violation**					
Yes	77	5.19%	42.86%	51.95%	100%
No	1533	6.65%	24.27%	69.08%	100%
*Road characteristics*					
**AADT (vehicle/day)**					
Less than or equal to 15,000	588	10.03%	28.57%	61.40%	100%
15,001–50,000	587	5.28%	23.68%	71.04%	100%
50,001–100,000	211	3.79%	25.12%	71.09%	100%
Over 100,000	224	3.57%	20.09%	76.34%	100%
**Alignment**					
Straight-level	1148	5.92%	24.13%	69.95%	100%
Straight-grade	240	7.50%	25.83%	66.67%	100%
Curve-level	94	10.64%	29.79%	59.57%	100%
Curve-grade	128	7.81%	29.69%	62.50%	100%
**Crash location**					
Highway Section	1103	6.71%	23.84%	69.45%	100%
Intersection	342	6.43%	30.70%	62.87%	100%
Ramp	43	4.65%	16.28%	79.07%	100%
Bridge	41	9.76%	31.71%	58.53%	100%
Other	81	4.94%	20.99%	74.07%	100%
**Divided road**					
Yes	884	5.66%	24.10%	70.24%	100%
No	726	7.71%	26.45%	65.84%	100%
**Number of lanes**					
Less than or equal to 2	478	10.88%	30.13%	58.99%	100%
Less than or equal to 4	717	4.88%	24.27%	70.85%	100%
Over 4	415	4.58%	20.96%	74.46%	100%
**Road surface condition**					
Dry	1246	7.06%	24.24%	68.70%	100%
Wet	271	4.43%	32.10%	63.47%	100%
Ice	86	6.98%	17.44%	75.58%	100%
Other	7	0.00%	14.29%	85.71%	100%
**Speed limit (mph)**					
Less than 30	76	0.00%	11.84%	88.16%	100%
30–45	438	3.88%	22.83%	73.29%	100%
46–55	470	9.36%	31.91%	58.73%	100%
56–65	450	6.67%	24.67%	68.66%	100%
over 66	176	8.52%	19.89%	71.59%	100%
**Road type**					
US route	346	7.80%	23.99%	68.21%	100%
Interstate	620	4.84%	21.94%	73.22%	100%
State route	453	7.51%	28.04%	64.45%	100%
Non-state route	191	7.85%	30.89%	61.26%	100%
**Setting**					
Urban	784	9.18%	26.66%	64.16%	100%
Rural	826	4.12%	23.73%	72.15%	100%
*Vehicle characteristics*					
**Gross weight (lbs.)**					
Less than or equal to 10,000	58	3.45%	15.52%	81.03%	100%
10,001–26,000	168	4.17%	26.79%	69.04%	100%
over 26,000	1384	7.01%	25.36%	67.63%	100%
**Vehicle type**					
Single-unit truck	389	5.14%	29.31%	65.55%	100%
Truck/trailer	199	5.53%	23.12%	71.35%	100%
Truck/tractor	24	8.33%	20.83%	70.84%	100%
Tractor/semi-trailer	758	8.71%	23.22%	68.07%	100%
Tractor/doubles	35	2.86%	25.71%	71.43%	100%
Other	205	2.93%	26.83%	70.24%	100%
**Vehicle defect**					
Yes	596	5.54%	21.64%	72.82%	100%
No	1014	7.20%	27.22%	65.58%	100%
**Number of vehicles**					
1	385	5.19%	24.68%	70.13%	100%
2	1093	5.95%	23.15%	70.90%	100%
3	98	14.29%	42.86%	42.85%	100%
Greater than or equal to 4	34	20.59%	44.12%	35.29%	100%
*Crash characteristics*					
**Collision type**					
Head-on	31	41.94%	38.71%	19.35%	100%
Rear-end	344	5.81%	35.47%	58.72%	100%
Angle	224	9.38%	30.80%	59.82%	100%
Sideswipe	433	3.70%	16.86%	79.44%	100%
Single vehicle	385	5.19%	24.68%	70.13%	100%
Other	193	8.29%	17.62%	74.09%	100%
**Crash hour**					
12:00–5:59 a.m.0:00–5:59	174	8.62%	31.03%	60.35%	100%
6:00–11:59 a.m.	569	7.03%	24.43%	68.54%	100%
12:00–5:59 p.m.	631	5.86%	25.20%	68.94%	100%
6:00–11:59 p.m.	236	5.93%	22.46%	71.61%	100%
**Fire/explosion/spill**					
Yes	124	11.29%	37.90%	50.81%	100%
No	1486	6.19%	24.09%	69.72%	100%
*Environmental characteristics*					
**Lighting conditions**					
Daylight	1159	5.87%	24.50%	69.63%	100%
Dusk/dawn	72	12.50%	26.39%	61.11%	100%
Dark—street lights	123	4.88%	33.33%	61.79%	100%
Dark—no street lights	256	8.98%	23.83%	67.19%	100%
**Weather**					
Clear	1125	6.67%	23.91%	69.42%	100%
Cloudy	218	6.88%	29.36%	63.76%	100%
Rain	164	1.83%	30.49%	67.68%	100%
Fog	18	16.67%	38.89%	44.44%	100%
Snow	85	11.76%	17.65%	70.59%	100%

Bold and italic texts represent the five major categories. Bold texts represent the subcategories in each major group.

**Table 2:** Bayesian network probability inference results for HAZMAT road transportation crash severity.

Variables	Probabilities When Setting Evidence		
	Fatal and Severe Injury Crashes	Injury Crashes	No Injury Crashes
Proportion distribution	0.1239	0.2602	0.6158
*Driver characteristics*			
**Driver age**			
less than 25	0.2187	0.3348	0.4464
25–35	0.1423	0.2329	0.6248
36–45	0.1169	0.2760	0.6071
46–60	0.1058	0.2596	0.6345
over 60	0.1527	0.2472	0.6001
**Distraction**			
Yes	0.1620	0.2761	0.5619
No	0.1224	0.2596	0.6180
**Fatigue**			
Yes	0.2112	0.2865	0.5022
No	0.1234	0.2601	0.6165
**Improper operation**			
Yes	0.1240	0.2622	0.6225
No	0.1236	0.2539	0.6138
**Speeding**			
Yes	0.1572	0.3017	0.5410
No	0.1208	0.2563	0.6230
**Violation**			
Yes	0.1920	0.2877	0.5203
No	0.1205	0.2589	0.6206
*Road characteristics*			
**AADT (vehicle/day)**			
Less than or equal to 15,000	0.1201	0.2684	0.6114
15,001–50,000	0.1263	0.2445	0.6292
50,001–100,000	0.1271	0.2660	0.6069
over 100,000	0.1246	0.2745	0.6009
**Alignment**			
Straight-level	0.1210	0.2585	0.6204
Straight-grade	0.1232	0.2595	0.6174
Curve-level	0.1245	0.2604	0.6152
Curve-grade	0.1318	0.2683	0.5999
**Crash Location**			
Highway Section	0.1111	0.2492	0.6397
Intersection	0.1503	0.2896	0.5601
Ramp	0.1639	0.2786	0.5575
Bridge	0.1582	0.2548	0.5870
Other	0.1490	0.2791	0.5719
**Speed limit (mph)**			
less than 30	0.1399	0.1834	0.6767
30–45	0.1111	0.2273	0.6616
46–55	0.1216	0.3022	0.5761
56–65	0.1247	0.2732	0.6021
over 66	0.1533	0.2301	0.6166
*Vehicle characteristics*			
**Vehicle type**			
Single-unit truck	0.1223	0.2732	0.6045
Truck/trailer	0.1179	0.2494	0.6327
Truck/tractor	0.1317	0.2605	0.6078
Tractor/semi-trailer	0.1274	0.2529	0.6197
Tractor/doubles	0.1215	0.2636	0.6149
Other	0.1195	0.2726	0.6079
**Vehicle defect**			
Yes	0.1266	0.2557	0.6177
No	0.1223	0.2629	0.6147
**Number of vehicles**			
1	0.1149	0.2435	0.6416
2	0.1116	0.2512	0.6372
3	0.2357	0.3883	0.3760
Greater than or equal to 4	0.2995	0.3720	0.3285
*Crash characteristics*			
**Collision type**			
Head-on	0.3997	0.3028	0.2975
Rear-end	0.1157	0.3431	0.5412
Angle	0.1948	0.3311	0.4741
Sideswipe	0.0772	0.1954	0.7274
Single vehicle	0.0881	0.2325	0.6794
Other	0.1375	0.2223	0.6402
**Crash hour**			
12:00–5:59 a.m.	0.1736	0.2992	0.5272
6:00–11:59 a.m.	0.1133	0.2510	0.6357
12:00–5:59 p.m.	0.1061	0.2470	0.6469
6:00–11:59 p.m.	0.1605	0.2892	0.5503
**Fire/Explosion/Spill**			
Yes	0.2396	0.3347	0.4258
No	0.1143	0.2540	0.6317
*Environmental characteristics*			
**Lighting condition**			
Daylight	0.1016	0.2447	0.6537
Dusk/dawn	0.2239	0.2937	0.4823
Dark—street lights	0.1650	0.3425	0.4926
Dark—no street lights	0.1770	0.2818	0.5412
**Weather**			
Clear	0.1216	0.2592	0.6192
Cloudy	0.1259	0.2571	0.6170
Rain	0.1292	0.2634	0.6073
Fog	0.1294	0.2683	0.6023
Snow	0.1364	0.2700	0.5936

Bold and italic texts represent the five major categories. Bold texts represent the subcategories in each major group.

**Table 3:** Confusion matrix for the Bayesian network.

Confusion Matrix		Predicted				
		Class 1	Class 2	Class 3	False Negative (FN)	Sensitivity
**Actual**	Class 1	T_11_	F_21_	F_31_	F_21_ + F_31_	$\frac{T_{11}}{T_{11}{+F}_{21}{+F}_{31}}$
	Class 2	F_12_	T_22_	F_32_	F_12_ + F_32_	$\frac{T_{22}}{F_{12}{+T}_{22}{+F}_{32}}$
	Class 3	F_13_	F_23_	T_33_	F_13_ + F_23_	$\frac{T_{33}}{F_{13}{+F}_{23}{+T}_{33}}$
	False positive (FP)	F_12_ + F_13_	F_21_ + F_23_	F_31_ + F_32_	Overall Accuracy $\frac{T_{11}{+T}_{22}{+T}_{33}}{T_{11}{+T}_{22}{+T}_{33}{+F}_{21}{+F}_{31}{+F}_{12}{+F}_{32}{+F}_{13}{+F}_{23}}$	
	Precision	$\frac{T_{11}}{T_{11}{+F}_{12}{+F}_{13}}$	$\frac{T_{22}}{F_{21}{+T}_{22}{+F}_{23}}$	$\frac{T_{33}}{F_{31}{+F}_{32}{+T}_{33}}$		

**Table 4:** Performance measurements for the Bayesian network model.

Performance Measurements	Fatal and Severe Injury Crashes	Injury Crashes	No Injury Crashes
Accuracy	96.4%	85.9%	85.0%
Precision	67.5%	78.5%	88.1%
Sensitivity	82.4%	69.4%	89.8%
Specificity	97.1%	92.4%	75.4%
F-score	74.2%	73.7%	88.9%
Overall accuracy = 85.8%			

## Data Availability

The data used in this study are from the Highway Safety Information System (HSIS), which can be requested at https://www.hsisinfo.org (accessed on 22 November 2021).
